# Massachusetts Regulations

**Published:** 1894-12

**Authors:** 


### MASSACHUSETTS REGULATIONS.

Bearing date of November 5th, the Board of Cattle Commissioners of the Commonwealth of Massachusetts have issued a manifesto containing the following rules:

1st. Quarantine regulations upon cattle entering from without the borders of the Commonwealth.

2d. Regulation of cattle traffic at Brighton, Watertown and Somerville, which shall include all animals from within and without the Commonwealth.

3d. Systematic inspection of all herds in the State, beginning at the Cape; followed by extermination of diseased animals, disinfection of contaminated premises, and fixed quarantine regulations.

Their endorsement of the value of tuberculin as a diagnostic agent, is promulgated in the three following statements:

1st. That tuberculin is a reliable agent for determining the presence of tuberculosis in cattle.

2d. That tuberculin, properly prepared and carefully handled, can have no injurious effect upon healthy animals.

3d. That it is the only known means whereby a positive diagnosis can be made in the early stages of the disease.

In General Order, No. 3, they have quarantined against the United States, District of Columbia, Canada, Great Britain and any other localities, and declare the same to be infected districts. All animals entering the State after November 20th will be subject to quarantine until they have been inspected and released under order of said Board, except such animals as might be destined for foreign shipment, or intended for preparation as dressed beef for foreign shipment, and in the event of an accident by which it might be necessary to temporarily unload animals within the borders of said Commonwealth. All animals affected with tuberculosis are to be condemned and slaughtered by the act establishing said Board.

General Order, No. 4, fixes Brighton, Watertown and Somerville as quarantine stations to which all animals must be shipped, intended for use within the borders of the State. All railroads and common carriers are directed to take cognizance of General Order No. 3.

Under date of November 25th, all cattle in Nantucket County were ordered quarantined upon the premises of their owners, for the purpose of commencing their examination, with tuberculin, for the existence of tuberculosis. Compensation is to be allowed for all those condemned and destroyed. All free from the disease are branded with a seal, which makes them free to all markets in the State.

All premises will be disinfected, and this plan will be followed in Dukes and Barnstable Counties, and gradually extended throughout the State.

This movement is the most extensive one adopted by any State in the Union for dealing with this very important subject, and all eyes will be directed toward Massachusetts to note the results of the very thorough work they propose doing. We trust we may be favored from time to time by the Commissioners with brief accounts of the progress of their work, that through the columns of the Journal we may keep our readers informed, and thus strengthen the contemplated movements in many other States.

